# The Role of the Microbiome in Allergy, Asthma, and Occupational Lung Disease

**DOI:** 10.1007/s11882-024-01156-8

**Published:** 2024-06-21

**Authors:** Ashley Peer, Derrick R. Samuelson

**Affiliations:** 1 Department of Internal Medicine, Division of Pulmonary, Critical Care, and Sleep, University of Nebraska Medical Center, Omaha, NE USA; 2 Nebraska Food for Health Center, University of Nebraska-Lincoln, Lincoln, NE USA

**Keywords:** Microbiota, Lung disease, Allergy, Asthma, Exposures

## Abstract

**Purpose of Review:**

The human commensal microbiota is now widely accepted as a key regulator of human health and disease. The composition of the mucosal associated microbiota has been shown to play a critical role in the lung health. The role of the mucosal microbiota in the development and severity of allergy, asthma, and occupational lung disease is only beginning to take shape. However, advances in our understanding of these links have tremendous potential to led to new clinical interventions to reduce allergy, asthma, and occupational lung disease morbidity.

**Recent Findings:**

We review recent work describing the relationship and role of the commensal microbiota in the development of allergy, asthma, and occupational lung disease. Our review primarily focuses on occupational exposures and the effects of the microbiome, both in composition and function. Data generated from these studies may lead to the development of interventions targeted at establishing and maintaining a healthy microbiota. We also highlight the role of environmental exposures and the effects on the commensal microbial community and their potential association with occupational lung disease.

**Summary:**

This review explores the current research describing the role of the human microbiome in the regulation of pulmonary health and disease, with a specific focus on the role of the mucosal microbiota in the development of allergy, asthma, and occupational lung disease.

## Introduction

One of the very first descriptions of the microbiome dates to the late 1800s when Theodore Escherich published his thesis titled “The Intestinal Bacteria of the Infant and Their Relation to the Physiology of Digestion [1].” The study of the human microbiome has since undergone a massive paradigm shift. Within the past decade alone, more than US$1.7 billion has been allocated for human microbiome research [2], demonstrating just how imperative it is to gain a better understanding of the microbiome and how it interacts with its surroundings. Specifically, the meta-organism concept or holobiont theory has become universally accepted as numerous preclinical and clinical studies have demonstrated a critical role of commensal microorganisms in human health [3–14]. Understanding the mechanisms that mediate crosstalk between the mucosal microbial communities and the lungs and how this interaction facilitates optimal lung health is a rapidly growing area of research. The role of the GI microbiota on mediating, maintaining, and regulating the health of multi-organ systems is an expanding area of research that has massive potential to aid in the development of novel treatment and management strategies for lung disease.

## The Role of the Microbiome in the Development of Allergy

A vital role between various exposures and the composition of the mucosal microbiota and subsequent development of allergic disease has been established by multiple groups [14–28]. Specifically, maternal prenatal exposure to pets, vaginal vs. c-section modes of delivery, childhood environmental exposures, and childhood exposure to pets have all been associated with a reduced allergy risk. In addition, breastfeeding, early exposure to antibiotics and the development of oral tolerance to different antigens all play a critical role in the development of allergy. Importantly the dysregulation of resident microbial communities due to alterations in these processes may represent a key component that drives allergy risk**.** For instance, changes in the cutaneous microbiome poses a risk for atopic dermatitis (AD) flare-ups, giving biofilm-producing *Staphylococcus aureus* the opportunity to overgrow. Additionally, it was demonstrated that individuals with AD lack mucin-producing bacteria that provide sustenance for beneficial commensal gut microbes. In patients with allergic rhinitis (AR), dysbiosis of the nasal bacteriome is a novel rising area of interest. Furthermore, it has been established that patients with distinct food allergies have gut dysbiosis, leading to a decrease in short chain fatty acids (SCFAs). Interestingly, in all these conditions, residential microbiome colonization and antibiotic use are extraordinarily important during the first few years of life. Microbial transplants and supplementation with commensal microbes have shown promising results across various allergies. This topic has been subject to many review articles [14, 28–30], as such we will not focus on this topic. However, Table 1 provides an overview of the changes in the microbiota associated with the development of allergies [31–52].

**Table 1:**
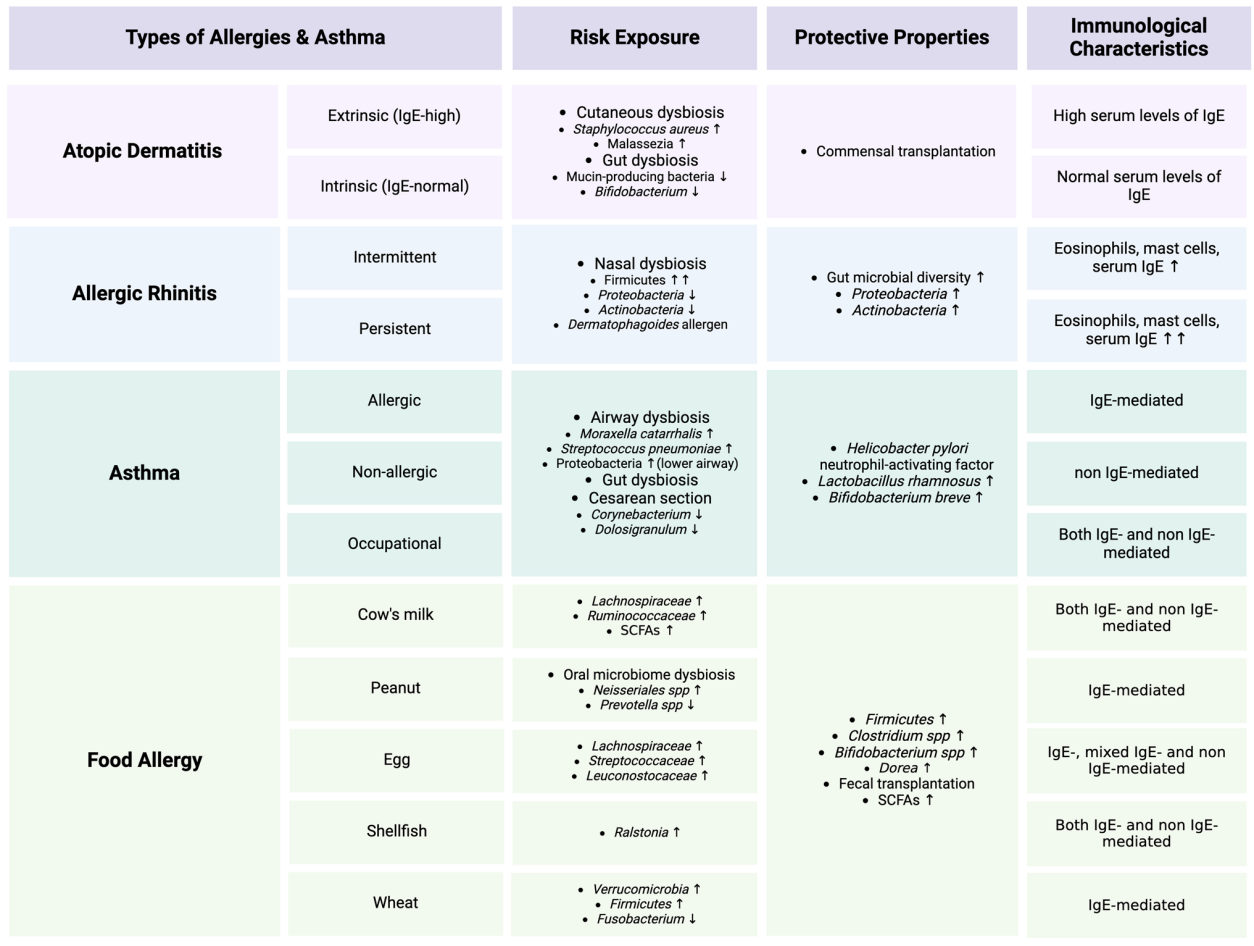
Microbial changes associated with allergies and asthma

## The Role of the Microbiome in the Development of Asthma

The composition of the GI and lung microbiota and the subsequent development of asthma is also well established [15, 16, 53–57]. Specifically, maternal prenatal exposure to pets, vaginal vs. c-section modes of delivery, childhood environments exposures, and childhood exposure to pets have all been associated with a reduced asthma risk. Particularly, dysbiosis of the airway can give rise to favorable growth conditions for *Proteobacteria* associated with viral respiratory infections. This dysbiosis dysregulates the crosstalk between the gut-lung axis and poses a serious risk for asthma development. Like allergy and the microbiome, the role of the microbiome in the development of asthma has been subject to many review articles [14, 15, 30, 55]. However, Table 1 provides an overview of the microbiota changes associated with the development of asthma [31–52].

## The Role of the Microbiome in Occupational Lung Disease

**Fig. 1:**
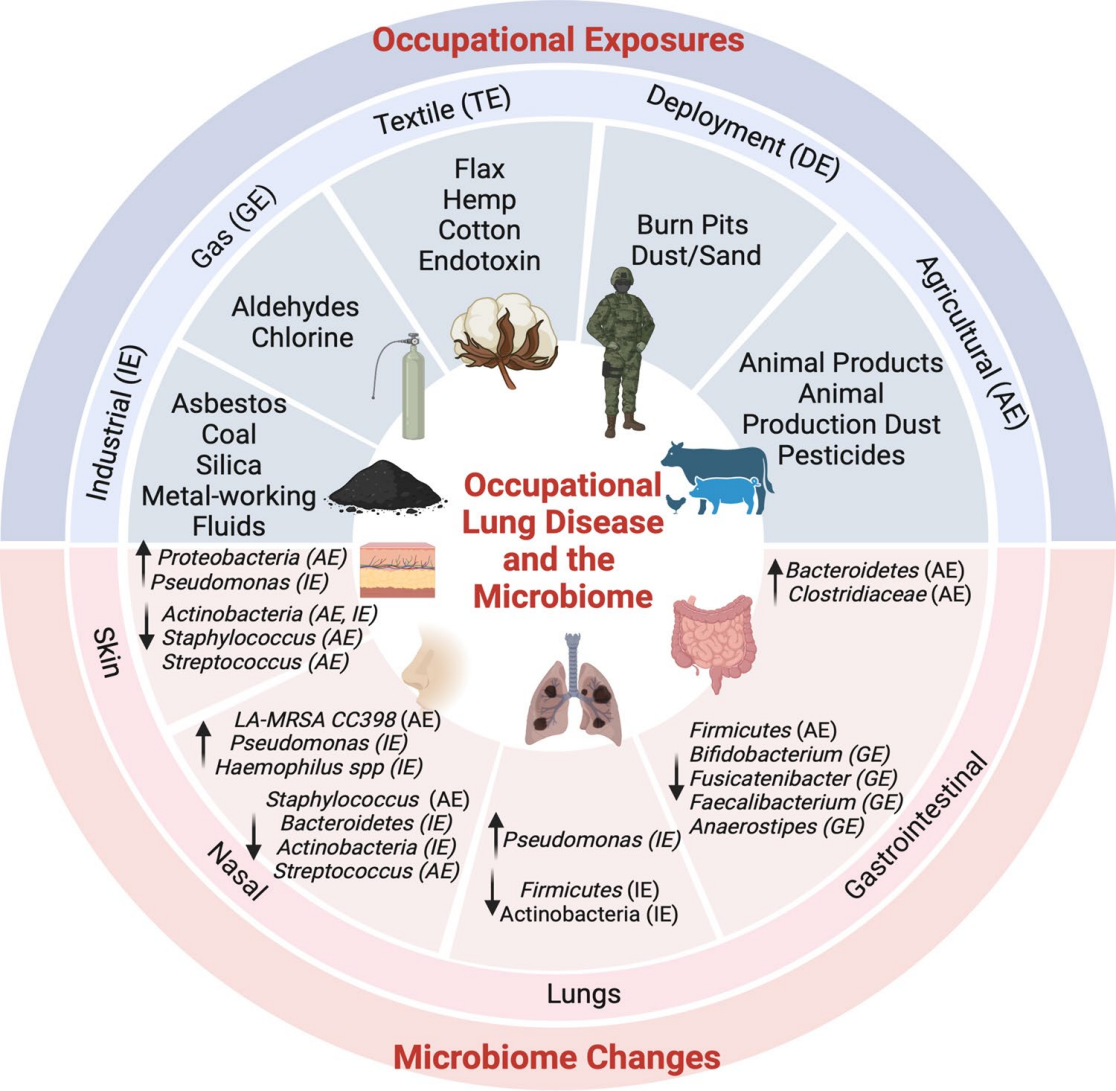
Changes in commensal microbial communities associated with occupational exposures

Our understanding of the composition of commensal microbiota and the subsequent development of occupational lung disease is limited. This section will review our current understanding of the effects of occupational exposures on the composition of the microbiota and their potential impact in the development of occupational lung disease. Figure 1 summarizes the current data regarding occupational exposures and the subsequent impact on commensal microbial populations.

## Agricultural Exposures and the Microbiome

### Animals/Animal Products

One of the best studied occupational exposures relative to the microbiome comes from studies involving agriculture workers exposed to various animals and animal products. Early studies demonstrated a significant association between bacterial communities in air samples from swine barns with the nasopharyngeal flora of the workers in contact with pigs. These similarities were not observed in non-exposed control groups [58]. Similarly, other studies demonstrated seasonal variability in the microbes comprising the air of pig barns and the correlation with fluctuation in the nasal microbiota of workers [59]. Additionally, shared microbial diversity seemed to be, in part, dependent on contact time with the animals, as the air-microbiota from pig farms and the nasal samples from pig farmers had 31.7% shared OTUs, which was significantly higher than the 23.4% shared OTUs between the bioaerosols in pig slaughterhouses and slaughterhouse workers [60]. Finally, other studies have demonstrated a higher similarity in gut microbiota between farmers and pigs, than between farmers and non-exposed human controls [61]. Additionally, pig farmers had less microbiota species diversity when compared to non-exposed villagers, which may suggest a higher health risk. These data were further confirmed by a longitudinal investigation of the impact of the swine farm on the gut microbial composition [62]. Specifically, veterinary students who had 3-month internships at a swine farm, exhibited a significant change their gut microbiota, which was observed to be more similar in composition to full-time farm workers’. Interestingly, these changes only partially reverted following 6-months of no exposure [63]. The composition of the nasal microbiome in subjects during and after long- and short-term exposure to livestock-associated MRSA showed similar trends in microbial imbalance. Nasal samples from all the tested pig farm workers showed the presence of LA-MRSA CC398. Further, all nasal samples collected immediately after a short-term exposure to the farm were positive for MRSA, which is particularly relevant as all the nasal samples collected prior to the farm visit were MRSA-negative [64]. In addition, to changes associated with swine operations, the species richness in the nasal microbiota of dairy farmers compared to non-dairy farmers was significantly higher. Further, dairy farmers had lower nasal carriage of *Staphylococcus spp* [65]. Additionally, higher levels of *Proteobacteria,* but lower levels of *Actinobacteria,* were observed on the forearm skin microbiota of farmworkers. Farm animal operations were also associated with a reduction in the prevalence of *Staphylococcus* and *Streptococcus* on the skin [66].

### Pesticides

The effects of agricultural pesticide exposure on the microbiome have only been assessed in one study. The microbial composition of buccal (oral) microbiota was found to be significantly associated with blood concentration of the insecticide azinphos methyl. Specifically, there was a significant reduction in the abundance of *Streptococcus* [67]. In addition to human studies, there has been one animal study investigating the relationship between intestinal dysbiosis and pesticide exposure. Using a rat model of chlorpyrifos exposure, researchers found that exposed rats had significant changes in their gut microbiota, as well as changes in the metabolic capacity of the microbiota, all of which lead to an increased incident of obesity when compared to nonexposed rats [68].

## Industrial Exposures and the Microbiome

### Metalworking Fluid Exposure

Metalworking fluid is a cooling and lubricating fluid that is frequently colonized by microorganisms such as *Pseudomonas.* Interestingly, lung biopsies from symptomatic (pulmonary symptoms) workers exposed to metalworking fluid demonstrated an increased abundance of bacteria species typically isolated from the metalworking fluid. The pulmonary condition associated with metalworking fluids was characterized by lymphocytic bronchiolitis and alveolar ductitis with B-cell follicles and emphysema [69]. All symptomatic workers had an increased abundance of *Pseudomonas* in the lung, skin, and nasal samples of exposed workers, which was also found in metalworking fluid, suggesting that pulmonary *Pseudomonas* may have been derived from the metal working fluid. The *Pseudomonas* isolate was not identified in air samples from the metalworking shops, which suggests that *Pseudomonas* is being transmitted via contact with the metalworking fluid and not the the air [70]. Several additional studies have evaluated the effects heavy metal exposure on the microbial composition of the GI tract. For example, in both human and animal studies arsenic exposure alters the gut microbiome [71, 72]. Specifically, a higher abundance of *Proteobacteria* was observed in children with high arsenic exposure when compared to the low arsenic exposure controls [72]. Furthermore, WGS sequencing demonstrated that genes involved in virulence and multidrug resistance were positively correlated with arsenic levels. In mice, exposure to 100 ppb arsenic for 13 weeks significantly alters the composition and functional capacity of the GI microbiota compared to controls [71].

### Dust Exposure

A limited number of papers have investigated the effects of dust (silica and ceramic dust) exposures on the microbiome. Subjects with early-stage pulmonary fibrosis due to silica exposure had significantly lower levels of *Firmicutes* and *Actinobacteria* in their GI microbial communities than healthy controls [73]. Further, the composition of the nasal microbiota in workers exposed to dust in ceramic factories was significantly altered. Specifically, there were marked increases in the abundance of *Haemophilus* spp., which was accompanied by a significant decrease in the abundance of *Actinobacteria* and *Bacteroidetes*, when compared to health non-exposed controls [74].

## Deployment Exposures and the Microbiome

### Chemical Exposure

Few studies have examined the microbiome in military personnel in relationship to deployment related exposures. One longitudinal study evaluated the respiratory tract microbial communities of healthy military personnel. This study found that *Staphylococcus*, *Corynebacterium*, and *Propionibacterium* accounted for ~75% of all microbial species in the nasal and nasopharyngeal microbiota, while *Streptococcus* was the only dominant bacterial genus in the oropharynx [75]. However, this study did not include a control group, which makes it difficult to evaluate if military exposures were associated with any changes in microbial composition. Similarly, in a mouse model of Gulf War illness, exposure to Gulf War chemicals were associated with GI microbial dysbiosis and decreased GI barrier function [76]. Interestingly, a follow-up study found that oral butyrate supplementation ameliorated many the associated effects of the chemical exposure in mice, suggesting that restoration of GI microbial functions may be an important factor in treating exposed personnel [77].

### Dust/Burn Pits Exposure

While a large ongoing longitudinal project (the US Veteran Microbiome Project, [78, 79]) has been collecting samples since 2015, no studies have investigated the relationship between military deployment related exposures and the microbiome to our knowledge.

## Chemical/Gas/Smoke Exposures and the Microbiome

### Gas Exposure During Occupational Diving

To date only one study has evaluated the effects of helium–oxygen saturation diving on the GI microbial community. Specifically, there was a marked decrease in the abundance of *Bifidobacterium Fusicatenibacter*, *Faecalibacterium*, and *Anaerostipes* following saturation diving. The prevalence of *Lactococcus*, *Actinomyces*, *Peptoclostridium*, *Butyricimonas*, *Streptococcus*, and *Porphyromonas* increased post-dive in comparison to samples collected pre-saturation diving [80].

### Smoke (Forest Fire, Wood Smoke) and Vehicle Emissions

Polychlorinated dibenzo-p-dioxins are formed and found naturally in the environment through volcanic eruptions and forest fires. Yet, the manufacturing of pesticides and burning organic materials *(i.e.,* garbage) are also a major source of occupational exposure and environmental contamination with dioxins. The two major dioxins that have been studied are 2,3,7,8-tetrachlorodibenzo-p-dioxin (TCDD) and 2,3,7,8-tetrachlorodibenzofuran (TCDF). To date we only have data from animal models and in vitro culture experiments on the effects of dioxin exposure on the microbiota. Specifically, conventional female mice treated with TCDD showed increased *Enterobacteriaceae*, as well as in increase in the reservoir of antibiotic-resistant genes when compared with control mice [81]. Finally, an in vitro study cultured human fecal suspensions with TCDD. Following treatment, fecal supernatants were incubated with intestinal epithelial cells and IL-8 secretion from intestinal epithelial cells was assessed [82]. TCDF exposure increased IL-8 only in the presence of microbial products, which suggest that dioxin-mediated dysbiosis may promote inflammation [82]. Similarly, rats exposed to smoke from either smoldered sawdust or motor vehicle exhaust over 4 weeks exhibited significance differences in the microbial diversity of the respiratory microbiota. Specifically, rats exposed to smoke or vehicle emissions exhibited a significant loss of *Proteobacteria* in the lungs, which correlated with significant changes in innate immune function [83]. Finally, mice exposed to concentrated ambient air (Chicago area) exhibited a significant increase in the diversity of the intestinal microbiota compared to controls. However, exposed mice exhibited a significant decrease in the abundance of *Firmicutes* [84].

## Textile Exposures and the Microbiome

Exposure to dust in textile factories (cotton and/or flax) was one of the first occupational exposures thought to be associated with lung disease, namely asthma and COPD. This is due to the fact that the highest levels of airborne endotoxin were historically found recorded in textile factories, such as cotton mills [85]. Following this observation, several research studies linked endotoxin in organic dust to both asthma and COPD development [86, 87]. However, the literature on inhaled endotoxin exposure has been incongruent. Environmental endotoxin exposure on the development of childhood asthma has been shown to be protective [56, 57], while other studies have observed either no effect [88] or even a harmful effect [89, 90]. To our knowledge, no studies have evaluated the effects of textile dust exposure or inhaled endotoxin on microbiota diversity.

## Conclusions

While the evidence for microbiome-mediated effects on the severity and development of allergy and asthma continue to grow, there remains a paucity of data regarding the functional consequences of microbial dysbiosis associated with occupational exposure. Data from both human and animal studies clearly describe marked differences in the composition, function, and immune phenotype of the microbial communities from multiple body sites following occupational exposure. Yet, to our knowledge, no studies have examined the functional consequences of the changes in the microbial communities with the development of occupational lung disease or occupational diseases in general. These knowledge gaps are an area primed for new investigation and will most likely significantly enhance our understanding of occupational exposures and the subsequent development of disease.

## Data Availability

No datasets were generated or analysed during the current study.
